# Relationship between autonomic dysfunction and sexual dysfunctions in Parkinson’s patients

**DOI:** 10.3389/fnagi.2025.1562003

**Published:** 2025-03-12

**Authors:** Hatice Varlıbaş, Hacı Ali Erdoğan, Ibrahim Acir, Vildan Yayla

**Affiliations:** ^1^Kırıkhan State Hospital, Hatay, Türkiye; ^2^Bakırköy Sadi Konuk Training and Research Hospital, Istanbul, Türkiye

**Keywords:** Parkinson’s disease, autonomic dysfunction, sexual dysfunction, non-motor, ASEX

## Abstract

**Objective:**

Parkinson’s disease (PD) is a neurodegenerative disorder characterized by motor and non-motor symptoms. Autonomic dysfunction, one of the non-motor symptoms, affects various systems such as the gastrointestinal, cardiovascular, genitourinary, and thermoregulatory systems. Sexual dysfunction (SD), however, is a frequently neglected issue in Parkinson’s patients. This study aimed to investigate the relationship between SD, findings of autonomic dysfunction in other systems, and the severity of PD.

**Methods:**

The study included 41 male and 35 female patients diagnosed with definitive idiopathic PD, with Hoehn and Yahr stages between 1 and 3, and without a diagnosis of diabetes or cognitive impairment. Demographic characteristics and disease duration of the patients were recorded. The following assessments were administered to the patients: Unified Parkinson’s Disease Rating Scale (UPDRS), Hoehn and Yahr Scale, Beck Depression Inventory (BDI), SCOPA-AUT questionnaire (Scales for Outcomes in Parkinson’s Disease Autonomic Dysfunction), short version of the QUIP (Questionnaire for Impulsive-Compulsive Disorders in Parkinson’s Disease), and ASEX (Arizona Sexual Experiences Scale).

**Results:**

The patients were divided into two groups: those with SD (53.9%) and those without SD (46.1%). Patients with SD had significantly higher age, PD stage, total SCOPA-AUT scores, and subdomain scores related to the cardiovascular, urinary, and gastrointestinal systems compared to those without SD (*p* < 0.001). The prevalence of hypertension was also significantly higher in the SD group (*p* = 0.001). An increase in UPDRS scores and depression severity, as measured by the Beck Depression Inventory, was associated with higher ASEX scores (*p* < 0.001). The frequency of impulse control disorder (ICD) was 6.5%; no significant differences were observed between patients with and without ICD in terms of equivalent levodopa dose or age (*p* = 0.58, *p* = 0.76).

**Conclusion:**

Although the presence of sexual dysfunction in Parkinson’s disease and its negative impact on quality of life have been recognized for many years, it is often overlooked for various reasons. The significant relationship identified in our study between SD, the severity of autonomic dysfunction, and disease stage may raise awareness of the early recognition of SD in PD patients. This could help prevent the neglect of this important non-motor symptom in disease management.

## Introduction

Parkinson’s disease (PD) is the second most common neurodegenerative disorder after Alzheimer’s disease, primarily characterized by motor symptoms such as bradykinesia, rigidity, tremor, and postural instability. However, non-motor symptoms, including autonomic dysfunction, significantly impact quality of life. Autonomic dysfunction in PD affects multiple systems, including the gastrointestinal, cardiovascular, genitourinary, and thermoregulatory systems, contributing to complications such as orthostatic hypotension, constipation, urinary disturbances, and impaired sexual function (Bloem et al., 2021; Dickson, 2018). Among these, sexual dysfunction (SD) remains underrecognized despite its prevalence and impact on well-being. Misconceptions and societal stigma often lead to its neglect, yet studies indicate that SD affects up to 80% of male and 85% of female PD patients—significantly higher than in age-matched controls (Bronner and Vodusek, 2011). Identifying and addressing SD as part of autonomic dysfunction could improve patient outcomes and quality of life. This study aims to explore the relationship between disease stage, autonomic dysfunction, and SD in PD patients.

## Methods

In this study, conducted between December 5, 2022, and February 5, 2023, a total of 76 patients (45 males and 31 females) diagnosed with idiopathic Parkinson’s Disease were prospectively evaluated.

Patients over 18 years of age, diagnosed with idiopathic PD according to the UK Parkinson’s Disease Society Brain Bank diagnostic criteria, with a disease duration of six months or more, at Hoehn and Yahr stages 1 to 3, and fluent in Turkish were included in the study. Exclusion criteria included spinal cord injuries or other neurological diseases, the diagnosis of urological conditions that may cause sexual dysfunction or drug use, diabetes, and a Brief Mental State Examination score below 24.

Various scales were used to determine the degree of disease progression and symptom severity in Parkinson’s disease. These included the MDS-Unified Parkinson’s Disease Rating Scale (MDS-UPDRS) to assess clinical severity, the Hoehn and Yahr Scale (HY) to evaluate clinical staging, and the International Classification of Functioning, Disability, and Health (ICF) to assess disability (Goetz et al., 2007; Goetz et al., 2004; Acır et al., 2020). In this study, the MDS-UPDRS and Hoehn and Yahr scales were administered to evaluate disease severity, while the SCOPA-AUT questionnaire (Scales for Outcomes in Parkinson’s Disease Autonomic Dysfunction) was used to assess the severity of autonomic dysfunction. The short version of the QUIP (Questionnaire for Impulsive-Compulsive Disorders in Parkinson’s Disease) test was employed to detect impulse control disorders (Visser et al., 2004; Weintraub et al., 2009). The Beck Depression Inventory was administered to identify depressive symptoms that may contribute to sexual dysfunction (Richter et al., 1998). The Arizona Sexual Experiences Scale (ASEX) was used to assess the presence of sexual dysfunction, with a total score of 19 or higher, or a score of 5 or more on any single item, indicating sexual dysfunction (McGahuey et al., 2000).

Statistical analyses were performed using SPSS version [X] (or other software if applicable). Descriptive statistics were presented as mean ± standard deviation (SD) for normally distributed variables and median [interquartile range] for non-normally distributed variables. The Shapiro–Wilk test was used to assess the normality of continuous variables.

Based on the distribution of the data, Pearson’s correlation coefficient was applied for normally distributed variables, while Spearman’s rank correlation was used for non-normally distributed data. Given that SCOPA-AUT and ASEX scores were not normally distributed, Spearman’s correlation analysis was performed to evaluate the association between autonomic dysfunction and sexual dysfunction. A *p*-value of <0.05 was considered statistically significant.

Additionally, multivariate regression analysis was conducted to examine the independent effects of autonomic dysfunction and disease severity on sexual dysfunction, adjusting for potential confounders such as age, disease duration, and depression severity (BDI scores). Assumptions of regression analysis, including linearity, homoscedasticity, and multicollinearity, were checked before model application.

## Results

A total of 76 patients diagnosed with idiopathic Parkinson’s disease participated in the study. The mean age of all patients was 64.8 years, with an average age of PD onset of 60.3 years and a mean disease duration of 4.45 years. Among male patients, 11 had a diagnosis of benign prostatic hyperplasia (BPH), while 17 patients had hyperlipidemia (HL), 19 had coronary artery disease (CAD), and 45 had hypertension (HT).

The mean total UPDRS score of the patients included in the study was 22.1, while the mean total SCOPA-AUT score was 16.9. The highest SCOPA-AUT scores were observed in the sections assessing symptoms related to gastrointestinal, urological, and sexual dysfunction.

The total MDS-UPDRS scores of the patients were compared with the total SCOPA-AUT scores using the Spearman correlation method. It was determined that as the total MDS-UPDRS score and subdomain scores increased, the total SCOPA-AUT score and the total scores of all subdomains, except for the pupillomotor system, showed a significant increase (*p* < 0.001).

Among them, 41 patients (53.3%) were categorized as having sexual dysfunction (SD), while 35 patients (46.7%) did not. Patients with SD were significantly older (mean: 70 years) than those without SD (mean: 59 years, *p* < 0.001). The mean age at disease onset was also later in patients with SD (65 years) compared to those without SD (55 years, *p* < 0.001). Hypertension was significantly more prevalent in the SD group (57.1%) than in the non-SD group (26.8%, *p* = 0.014). No significant differences were observed in the use of dopaminergic treatments (levodopa, pramipexole) between the two groups (*p* > 0.05), indicating that treatment use was not a major factor contributing to the observed differences in sexual dysfunction (Table 1).

**Table 1:** Distribution of SCOPA-AUT scores among patients with and without sexual dysfunction.

	SD - PD	SD + PD	*p*	*N*
	*N = 35*	*N = 41*		
Male	21 (60.0%)	24 (58.5%)	1	76
Female	14 (40.0%)	17 (41.5%)		
Age	59.0 [53.5; 64.5]	70.0 [66.0; 74.0]	<0.001	76
PD onset age	55.0 [51.5; 61.5]	65.0 [60.0; 68.0]	<0.001	76
Total duration of PD	3.00 [2.00; 5.00]	4.00 [2.00; 6.00]	0.232	76
Hypertension	20 (57.1%)	11 (26.8%)	0.014	76
Hoehn Yahr stages			<0.001	76
1–1.5	16 (45.6%)	2 (4.88%)		
2	19 (54.3%)	20 (48.8%)		
2.5–3	0 (0.00%)	19 (46.36%)		
UPDRS-Total	15.0 [12.5; 17.0]	26.0 [23.0; 36.0]	<0.001	76
SCOPA AUT- GI tract	3.00 [2.00; 4.00]	6.00 [6.00; 8.00]	<0.001	76
SCOPA AUT- Urinary	4.00 [2.00; 4.50]	6.00 [6.00; 9.00]	<0.001	76
SCOPA AUT- CVS	0.00 [0.00; 0.50]	2.00 [1.00; 4.00]	<0.001	76
SCOPA AUT- THERM	0.00 [0.00; 1.00]	1.00 [0.00; 2.00]	0.011	76
SCOPA AUT-Pupillomotor	0.00 [0.00; 0.00]	0.00 [0.00; 0.00]	0.520	76
SCOPA AUT-SD	2.00 [1.00; 2.00]	6.00 [5.00; 6.00]	<0.001	76
SCOPA AUT-Total	10.0 [7.00; 12.0]	22.0 [20.0; 27.0]	<0.001	76
BDI	6.00 [4.50; 8.00]	18.00 [13.0; 20.0]	<0.001	76

This figure illustrates the distribution of SCOPA-AUT total scores and subdomain scores (gastrointestinal, urinary, cardiovascular, thermoregulatory, and sexual dysfunction) in PD patients with and without sexual dysfunction (SD). Patients with SD exhibited significantly higher SCOPA-AUT total and subdomain scores, particularly in the gastrointestinal and urinary systems (*p* < 0.001), suggesting a strong correlation between autonomic dysfunction and SD severity.

SCOPA-AUT scores were significantly higher in patients with SD (22.0 ± 9.35) compared to those without SD (10.0 ± 7.0, *p* < 0.001), indicating more severe autonomic dysfunction in the SD group. SCOPA-AUT subdomains related to gastrointestinal, urinary, and cardiovascular dysfunction were particularly elevated in patients with SD. Gastrointestinal (5.20 ± 3.37) and urinary dysfunction (5.45 ± 3.26) were the most prevalent, emphasizing the involvement of these systems in autonomic dysfunction. Thermoregulatory dysfunction also showed significant differences between the two groups (*p* = 0.011).

The total ASEX score was significantly higher in patients with SD (24.0 ± 6.1) compared to those without SD (10.5 ± 3.6, *p* < 0.001), reflecting greater severity of sexual dysfunction in the SD group. The highest ASEX scores were observed in the items assessing orgasm (mean: 4.09) and pain during intercourse (mean: 3.89), highlighting common issues in these sexual domains.

The Beck’s Depression Inventory (BDI) scores were significantly higher in the group with sexual dysfunction (SD) compared to the group without SD (*p* < 0.001). In the correlation analysis between BDI and ASEX scores, it was observed that as BDI scores increased, both the total ASEX score and subitem scores significantly increased (*p* < 0.001).

A significant positive correlation was found between SCOPA-AUT scores and ASEX scores (*r* = 0.74, *p* < 0.001), reinforcing the association between autonomic dysfunction and sexual dysfunction (Table 2).

**Table 2:** Correlation between ASEX scores and SCOPA-AUT total scores.

	ASEX item-1	ASEX item-2	ASEX item-3	ASEX item-4	ASEX item−5	ASEX total
SCOPA AUT - GI tract	0.69/0.001<	0.71/0.001<	0.68/0.001<	0.69/0.001<	0.63/0.001<	0.74/0.001<
SCOPA AUT - Urinary	0.50/0.001<	0.50/0.001<	0.64/0.001<	0.62/0.001<	0.54/0.001<	0.59/0.001<
SCOPA AUT - CVS	0.52/0.001<	0.53/0.001<	0.61/0.001<	0.61/0.001<	0.54/0.001<	0.60/0.001<
SCOPA AUT - THERM	0.36/0.001	0.37/0.001	0.33/0.003	0.32/0.005	0.37/0.001	0.39/0.001<
SCOPA AUT - Pupillomotor	-0.01/0.948	-0.01/0.938	-0.01/0.931	-0.12/0.314	-0.05/0.679	-0.05/0.670
SCOPA AUT - SD	0.81/0.001<	0.80/0.001<	0.88/0.001<	0.85/0.001<	0.81/0.001<	0.90/0.001<
SCOPA AUT - Total	0.79/0.001<	0.79/0.001<	0.86/0.001<	0.82/0.001<	0.77/0.001<	0.87/0.001<

This scatter plot demonstrates the relationship between ASEX total scores (sexual dysfunction severity) and SCOPA-AUT total scores (autonomic dysfunction severity). A strong positive correlation (*r* = 0.74, *p* < 0.001) is observed, indicating that as autonomic dysfunction worsens, sexual dysfunction severity increases. These findings suggest that autonomic impairment is a key contributor to SD in PD patients.

As autonomic dysfunction worsened, the severity of sexual dysfunction increased. This was particularly evident in the gastrointestinal, urinary, and cardiovascular dysfunction subdomains, which exhibited the strongest correlations with sexual dysfunction.

No significant differences were observed in the equivalent levodopa dose between those experiencing impulse control disorders (ICD) and those without (*p* = 0.76). However, all patients with ICD were receiving dopamine agonists, suggesting a potential role for these medications in ICD development.

## Discussion and conclusion

Autonomic dysfunction, a common but underrecognized non-motor symptom of PD, significantly affects quality of life. In our study, most patients reported symptoms, with sexual dysfunction being a prominent yet understudied issue. These findings emphasize the importance of addressing non-motor symptoms alongside motor symptoms to improve the holistic care of PD patients.

In studies investigating the prevalence of autonomic dysfunction, a non-motor symptom of Parkinson’s disease, rates have been reported to range from 14 to 80% (Bloem et al., 2021; Jost, 2003). In our study, patients described autonomic symptoms in at least one system, while their most frequent and severe complaints were reported in items related to urinary, gastrointestinal system and sexual dysfunction.

Sexual dysfunction, however, is a less-studied aspect of autonomic involvement compared to other systems, and its diagnosis and treatment are often overlooked. Sexual dysfunction is a common component of the autonomic symptoms of Parkinson’s disease (PD). Sexual dysfunctions in Parkinson’s patients range from symptoms based on hyposexuality to hypersexuality associated with impulse control disorders. It is thought that the dopamine deficiency caused by dopaminergic neuron damage in Parkinson’s patients leads to symptoms of hyposexuality (Singer et al., 1991). Dopamine release in the medial preoptic area is known to support genital reflexes and sexual motivation (Dominguez and Hull, 2005; Hull and Dominguez, 2006). Animal studies have shown that stimulation of dopamine receptors (primarily D2 to D4 subtypes) in the paraventricular nucleus induces oxytocin release, leading to pro-erectile effects (Succu et al., 2007). Dopamine deficiency may also lead to sexual dysfunction by increasing the levels of the prolactin hormone, which negatively impacts libido (Krüger et al., 2005).

In addition to these mechanisms, motor symptoms of PD, such as tremor, rigidity, and bradykinesia, commonly affect sexual performance negatively. Furthermore, symptoms such as increased salivation and excessive sweating, seen in some patients, may reduce self-confidence and contribute to the development of sexual dysfunction.

The link between autonomic dysfunction, sexual dysfunction, and Parkinson’s disease (PD) progression involves multiple mechanisms. Dopaminergic deficiency, a hallmark of PD, disrupts sexual function by impairing dopamine’s role in libido, arousal, and orgasm regulation. While reduced dopamine levels can cause hyposexuality, dopamine agonists may lead to hypersexuality and impulse control disorders (Politis and Loane, 2011). Autonomic nervous system (ANS) dysfunction further exacerbates sexual dysfunction by affecting cardiovascular and genitourinary functions, leading to erectile dysfunction (ED) in men and dyspareunia in women due to impaired vascular and neural regulation.

Hormonal imbalances and neuroinflammation also contribute to sexual dysfunction in PD. Increased prolactin levels and disruptions in testosterone and estrogen suppress libido, while chronic inflammation and oxidative stress accelerate neurodegeneration and vascular dysfunction (David et al., 2024). Psychological factors, particularly depression and anxiety, further worsen sexual dysfunction, as lower serotonin and norepinephrine levels negatively affect desire and arousal. The observed correlation between higher Beck Depression Inventory (BDI) scores and sexual dysfunction highlights the impact of mood disorders on sexual health in PD (Reijnders et al., 2008).

Studies conducted on Parkinson’s patients have shown that the prevalence of sexual dysfunction (SD) is higher compared to the healthy population, with reported rates ranging from 42.6 to 79% in men and 36 to 87.5% in women (Brown et al., 1978).

Our findings indicate a higher prevalence of sexual dysfunction in Parkinson’s disease (PD) patients compared to the general aging population. While large-scale studies, such as the NSHAP and the English Longitudinal Study of Aging, demonstrate that sexual activity declines with age, they also highlight that healthy individuals maintain sexual interest and function to a greater extent than those with chronic illnesses (Jackson et al., 2020). In our study, 53.3% of men and 54.8% of women reported sexual dysfunction, suggesting that PD-related factors accelerate this decline beyond normal aging. Unlike in healthy individuals, where hormonal changes and psychological factors primarily contribute to sexual dysfunction, PD patients experience a multifaceted interplay of dopaminergic deficiency, autonomic failure, and motor impairments. Additionally, gender differences observed in PD parallel those in the general population, with men more commonly experiencing erectile dysfunction due to neurovascular impairment, while women face dyspareunia and anorgasmia, likely exacerbated by autonomic dysfunction. These findings underscore the need for targeted interventions addressing both neurological and psychosocial aspects of sexual health in PD patients.

Recent studies have further elucidated the relationship between autonomic dysfunction and sexual dysfunction (SD) in Parkinson’s disease (PD). A 2023 longitudinal study demonstrated that autonomic dysfunction is prevalent in early PD and progressively worsens over the first seven years, underscoring the need for early intervention. Additionally, research indicates that SD often precedes motor symptoms and remains under-recognized, highlighting the importance of early detection and management. These findings align with previous observations that autonomic dysfunction significantly impacts sexual health in PD patients.

This study highlights the significant association between autonomic dysfunction, sexual dysfunction (SD), and Parkinson’s disease (PD) severity. Our findings indicate a strong correlation between SCOPA-AUT total scores and ASEX scores (*r* = 0.74, *p* < 0.001), suggesting that worsening autonomic impairment contributes to more severe SD.

Our findings highlight a strong association between autonomic dysfunction, sexual dysfunction (SD), and Parkinson’s disease (PD) severity, emphasizing the need for comprehensive non-motor symptom management. The correlation between SCOPA-AUT and ASEX scores suggests that worsening autonomic failure directly contributes to SD, likely through dopaminergic and autonomic nervous system impairment.

Dopaminergic dysfunction disrupts mesolimbic pathways, reducing libido, while noradrenergic deficits affect autonomic regulation, contributing to erectile dysfunction and vaginal dryness. Prolactin elevation due to dopamine loss may further suppress sexual desire. Compared to other neurodegenerative diseases, SD in PD is primarily linked to autonomic dysfunction, whereas in Alzheimer’s disease (AD) it results from cognitive decline, and in multiple system atrophy (MSA) it appears earlier due to severe autonomic failure.

Clinically, these findings underscore the importance of routine SD screening in PD patients. Pharmacological interventions (dopamine adjustments, PDE5 inhibitors, hormone therapy) and psychological support (CBT, counseling) should be integrated into patient care. Future research should explore longitudinal SD progression and targeted autonomic interventions to improve quality of life in PD patients.

Many studies have investigated the effects of advancing age and disease severity on sexual function in Parkinson’s disease. In an Italy-based study, the frequency of sexual dysfunction was found to be 57%, and a significant relationship was reported between SD, high UPDRS scores, and the severity of depression (Raciti et al., 2020). Another study, in which ASEX total scores and subscales were significantly higher in Parkinson’s patients, observed a significant correlation between SD and the severity of PD (Elshamy et al., 2021). In a study conducted on male Parkinson’s patients, the frequency of erectile dysfunction was reported as 70%, and it was shown that increasing age was associated with greater severity of sexual dysfunction (Shalash et al., 2020).

In our study, patient age, Hoehn and Yahr stage, as well as total and subgroup scores of UPDRS, were found to be significantly higher in the patient group with SD. The data obtained support the conclusion that the frequency and severity of sexual dysfunction symptoms increase with the progression of Parkinson’s disease. It is suggested that this may be related to the worsening of systemic autonomic dysfunction as the disease stage advances.

Ozcan et al. reported a correlation between disease duration (mean disease duration of 6.3 years) and ASEX scores (Özcan et al., 2014). Similarly, Wermuth et al. found a relationship between the duration of the disease (mean disease duration of 5.4 years) and the frequency of sexual dysfunction (Wermuth and Stenager, 1995). However, in our study, no significant difference was observed between the mean disease duration of the groups with and without SD. This finding may be attributed to the relatively short mean disease duration in our patient group (mean disease duration: 4.45 years).

Conflicting results have been reported in studies evaluating the relationship between gender and sexual dysfunction (SD) in Parkinson’s disease. Zhao et al. found that the incidence of SD was higher in men with Parkinson’s disease compared to controls, while no significant increase was observed in female patients compared to the control group (Zhao et al., 2019). Conversely, in a study focusing exclusively on female Parkinson’s patients, it was reported that sexual dysfunction was significantly higher in women with Parkinson’s disease than in healthy controls (Varanda et al., 2016).

In our study, the frequency of sexual dysfunction was found to be 53.3% in men and 54.8% in women, with no significant relationship observed between the frequency of SD and gender differences. However, the relatively small sample size and the absence of a control group limit the ability to draw definitive conclusions regarding the relationship between gender and SD.

Studies investigating the subtypes of sexual dysfunction in Parkinson’s patients have reported that erectile dysfunction and difficulty reaching orgasm are among the most common issues. Bronner et al. determined that while difficulty with arousal and reaching orgasm was more prevalent in women, erectile dysfunction, sexual dissatisfaction, and premature ejaculation were the primary complaints in men (Bronner et al., 2004). In the PRISM cohort, difficulty reaching orgasm was also more common in female Parkinson’s patients, whereas erectile dysfunction was more frequently observed in males (Kinateder et al., 2022). Similarly, in our study, vaginal dryness and difficulty reaching orgasm were prominent in women, while erectile dysfunction and premature ejaculation were more common in men.

Mood disorders may contribute to the development of sexual dysfunction (SD) in Parkinson’s patients. In a study investigating the relationship between SD and mood disorders in Parkinson’s patients, ASEX total scores were found to correlate with age, Hoehn and Yahr stage, and Hamilton Anxiety Rating Scale scores (Özcan et al., 2014). In our study, the severity of depression was assessed using the Beck Depression Inventory, and a significant increase in depression severity was observed in the group with SD compared to the group without SD.

The relationship between hypertension (HT), antihypertensive drugs, and sexual dysfunction is a frequently studied topic. In animal experiments investigating the effects of hypertension on the genital system, it was found that HT causes structural changes in the penile vascular system due to increased angiotensin II levels (Tobllı, 2004). In addition to the primary effects of hypertension, many antihypertensive drugs, such as beta-blockers, thiazide diuretics, and angiotensin receptor inhibitors, are believed to contribute to erectile dysfunction (Doumas and Douma, 2006).

In our study, a significant relationship was observed between SD and the presence of HT. However, we believe that multicenter studies involving larger patient populations and control groups are needed to determine whether this relationship is attributable to HT itself or the antihypertensive medications used.

Hypersexuality, which can be observed in Parkinson’s patients, is a component of ICD, alongside behaviors such as binge eating, pathological gambling, and compulsive shopping. Dopamine replacement therapy is believed to contribute to the development of ICD by overstimulating the mesolimbic dopaminergic system (Weintraub and Nirenberg, 2012). In our study, 14.47% of the patients reported ICD symptoms in at least one area, with compulsive shopping being the most frequently reported symptom in both genders. All patients with ICD were found to be using dopamine agonists. However, no significant difference in the dopaminergic treatment dose (equivalent levodopa dose) was observed between the group with ICD and the group without ICD.

Several limitations should be considered when interpreting the results of this study. Firstly, the study’s cross- sectional design limits the establishment of causality between autonomic dysfunction, sexual dysfunction, and disease progression in Parkinson’s disease. Longitudinal studies are needed to elucidate the temporal relationships between these variables. Secondly, the study was conducted at a single center, which may limit the generalizability of the findings to broader PD populations. Future multi-center studies are warranted to validate the results across diverse patient cohorts. Additionally, the reliance on self-reported measures such as the ASEX and QUIP scales may introduce response bias and underreporting of symptoms. While our sample size is limited, our study has several strengths, including comprehensive assessment methods, exploration of multiple mechanisms, clinical relevance, novel contributions, and the inclusion of psychological factors. By utilizing SCOPA-AUT and ASEX scales, our findings enhance the understanding of sexual dysfunction in Parkinson’s disease and highlight the importance of early screening and intervention.

This study did not include laboratory assessments to evaluate potential endocrine and metabolic contributors to sexual dysfunction. Hormonal imbalances, including testosterone, prolactin, LH, and FSH levels, were not measured, limiting our ability to differentiate between PD-related autonomic dysfunction and hypogonadism as causes of SD. Additionally, thyroid function tests (TSH, T3, T4) were not conducted, leaving open the possibility of undiagnosed thyroid disorders influencing libido. Stress-related sexual dysfunction was also not assessed through cortisol levels, and the absence of PSA testing in men with BPH prevents ruling out prostate-related sexual dysfunction as a confounding factor (Bhasin et al., 2007). Finally, the exclusion of patients with certain comorbidities and cognitive impairments may have led to a selection bias, potentially affecting the external validity of the findings. These limitations should be considered when interpreting the study’s results and implications.

This study reveals the intricate interplay between autonomic dysfunction, sexual dysfunction, and disease progression in Parkinson’s disease. Addressing these non-motor symptoms alongside motor symptoms is crucial for enhancing patients’ overall quality of life. Early detection and tailored interventions targeting autonomic and sexual dysfunction are essential for optimizing treatment outcomes. Comprehensive management strategies should consider the multifaceted nature of PD, integrating approaches that address both motor and non-motor aspects. By emphasizing the importance of holistic care, this research contributes to improving the well-being of individuals living with Parkinson’s disease.

## Data Availability

The raw data supporting the conclusions of this article will be made available by the authors, without undue reservation.
